# A Cost-Effective Liquid Chromatography Method with Ultraviolet Detection for Identity Screening and Assay of Injectable Antibiotics

**DOI:** 10.3390/molecules30102151

**Published:** 2025-05-13

**Authors:** Haile Kassahun Desta, Gebremariam Ketema, Ann Van Schepdael, Erwin Adams

**Affiliations:** 1 Department of Pharmaceutical and Pharmacological Sciences, Pharmaceutical Analysis, KU Leuven, 3000 Leuven, Belgium; hailekassahun.desta@kuleuven.be (H.K.D.); ann.vanschepdael@kuleuven.be (A.V.S.); 2 Department of Pharmacy, College of Medicine and Health Sciences, Wollo University, Dessie P.O. Box 1145, Ethiopia; gmariam81@gmail.com

**Keywords:** injectable antibiotics, liquid chromatography, low-income countries, substandard medicines

## Abstract

The presence of substandard and falsified (SF) medicines poses a significant challenge in resource-limited countries. Low-quality antibiotics are commonly reported in low-income countries. The present study aimed to develop and validate a liquid chromatography method with ultraviolet detection (LC-UV) for the identity screening and assay of 13 different injectable antibiotics, i.e., cefepime, amoxicillin, cefazolin, ampicillin, chloramphenicol, ceftazidime, ceftriaxone, cefotaxime, vancomycin, flucloxacillin, cloxacillin, benzylpenicillin, and meropenem in pharmaceutical formulations. Separation was performed using an XBridge C18 column and gradient elution. Mixtures of acetonitrile and 20 mM phosphate buffer (pH 8.0) were used as the mobile phases. The screening method was validated in terms of specificity and robustness, while linearity, precision, accuracy, and sensitivity were checked for the quantification method. The determination coefficients (R^2^) following linear regression were all greater than 0.999. The method showed good precision, with relative standard deviation values below 1%. The percentage recovery values were close to 100%. The method was applied to analyze 17 injectable antibiotics collected from the Ethiopian market. All commercial samples analyzed contained the correct API and met USP content specifications.

## 1. Introduction

Bacterial infections remain a leading cause of morbidity and mortality worldwide, with higher burdens in low-income and middle-income countries (LMICs) [1,2,3]. Injectable antibiotics are often prescribed for the treatment of severe and life-threatening bacterial infections. Intravenously administered antibiotics are widely used in low-income countries [4,5]. Therefore, improving access to good-quality antimicrobial substances is crucial for effective treatment, lifesaving therapies, and reducing overall healthcare costs [6,7,8]. Quality assurance of medicines is vital to guarantee medicines reaching patients are safe, effective, and meet acceptable quality standards [9]. However, the presence of substandard and falsified (SF) medicines in developing countries adversely impacts patient safety, the national economy, and public confidence in the healthcare systems [10,11,12,13,14]. Injectable SF products can be particularly dangerous, leading to serious and potentially life-threatening risks to patients, including morbidity, mortality, treatment failure, poisoning, and adverse drug interactions. Moreover, the use of SF products may also lead to underdosing of antibiotics, which promotes the development and spread of antimicrobial resistance [7,15]. Ensuring the availability of safe and effective medicines within Africa’s healthcare system remains a significant challenge. Factors such as limited resources, weak border controls, inadequate analytical infrastructure, restricted laboratory capacities, and weak, fragmented health regulations have contributed to the proliferation of SF medicines [6,8,16].

Low-quality antibiotics are mostly reported in developing countries [10,17]. A meta-analysis in 2018 revealed that 12.4% of the antibiotics tested in LMICs were SF [7]. Substandard injectable ceftriaxone in Kenya [18], benzylpenicillin in Myanmar and Zimbabwe, cefepime in Pakistan, and ceftazidime in India, Mexico, the Philippines, and Vietnam have been reported [15]. According to a review by Kelesidis et al. [15], antibiotics with no active ingredients were the most common SF type, followed by antibiotics with too low contents of active ingredients. Poor-quality antimicrobial medicines significantly contribute to the global burden of infectious diseases [6,19].

**Figure 1:**
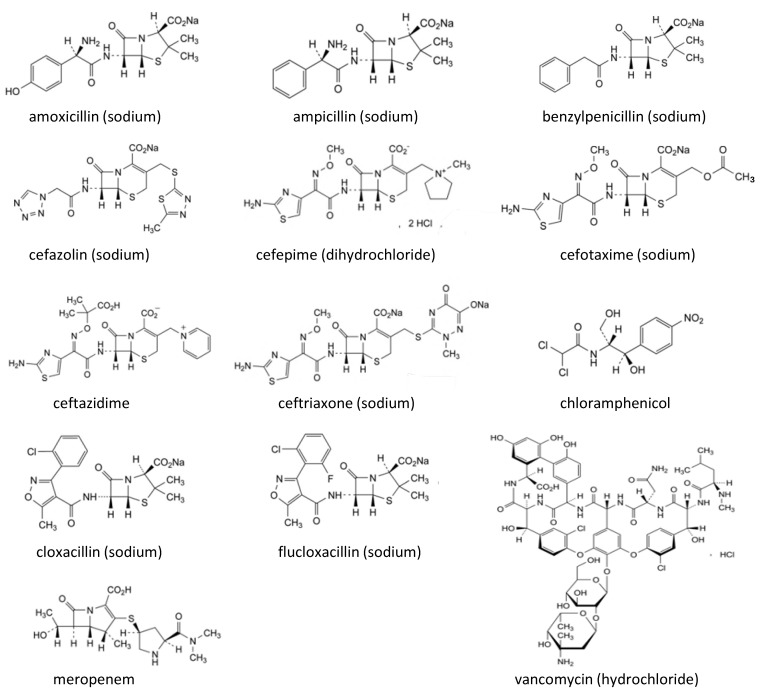
Chemical structures of the studied injectable antibiotics (adapted from [23]).

In the framework of fighting SF medicines in low-income countries, not only are regulatory efforts required, but also analytical methods are important. Chromatographic techniques are particularly valuable for this purpose. Pharmacopoeial monographs are each intended for a specific (injectable) antibiotic and require different mobile phases, columns, and experimental conditions [20]. Hence, implementing pharmacopoeial monograph testing for screening the identity and content is not very practical. Moreover, state-of-the-art techniques, like ultra-high performance liquid chromatography (UHPLC), eventually combined with mass spectrometry (MS), are mostly not affordable for laboratories in resource-limited countries. Therefore, simple and economical analytical methods are required for rapid screening of SF antimicrobial medicines [16,21]. In the present study, it was the intention to develop an easily applicable liquid chromatographic method with ultraviolet detection (LC-UV) for the identity screening and assay of 13 injectable antibiotics, i.e., cefepime, amoxicillin, cefazolin, ampicillin, chloramphenicol, ceftazidime, ceftriaxone, cefotaxime, vancomycin, flucloxacillin, cloxacillin, benzylpenicillin, and meropenem in pharmaceutical formulations. The samples were selected based on the literature, mainly their availability on the Ethiopian essential medicine list (EML) [22]. Chemical structures are shown in Figure 1. The development of a single method to analyze 13 injectable antibiotics avoids the need to set up a specific method for each drug, thus saving time and resources.

Until now, only a few chromatographic methods have been developed for the screening of antibiotics in pharmaceutical formulations. Mbinze et al. [24] reported an LC method to analyze different antibacterial agents. However, this method has been developed mostly for orally administered antimicrobial medicines, with only two injectable antibiotics (ceftriaxone and cefotaxime) included. Tie et al. [20] optimized a UHPLC-MS method for the identity screening and a UHPLC–diode array detection method for the quantification of some selected antimicrobial medicines. However, these methods were only applicable to a limited number of injectable antibiotics, including cefepime, cefazolin, ceftazidime, ceftriaxone, cefotaxime, and benzylpenicillin. Moreover, these analytical techniques are expensive and may not be easily available in resource-limited countries.

Therefore, there was a need to develop and validate an easy-to-use, accurate, and cost-effective LC-UV method for the quality control of 13 injectable antibiotics that have been commonly marketed in Ethiopia. Compared to already existing approaches, our method included a larger panel of antibiotics and used standard LC-UV equipment, making it particularly suitable for routine use in resource-limited quality control laboratories.

## 2. Results and Discussion

### 2.1. Method Development and Optimization

In developing countries, where the issue of SF medicines is significant, there is a need for methods that are accurate, affordable, and fast to ensure the safety and efficacy of essential medicines available on the market. In this study, an identity screening and quantification LC-UV method was developed and validated to analyze 13 injectable antibiotics commonly prescribed in developing countries. The screening method was designed to verify the presence or absence of active pharmaceutical ingredients (APIs) in the pharmaceutical formulations. This was achieved by comparing the retention times of sample peaks to those of the standards. The quantitative version was used to confirm that the correct amount of API was present in the formulations.

As a stationary phase, an XBridge C18 (250 mm × 4.6 mm, 5 µm) and Kinetex C18 column (150 mm × 4.6 mm, 2.6 µm) were tried. The XBridge C18 column was chosen because it is stable in both acidic and alkaline conditions. Hence, it can be used in a wide pH range (1–12). The Kinetex column was attempted because of the good results obtained for analyzing orally administered antimicrobial medicines in our previous work [25]. The initial chromatographic conditions were as follows: flow rate 1.0 mL/min and column temperature 30 °C. Detection was performed at 230 nm as all antibiotics showed UV absorbance at this wavelength. Using acetonitrile (ACN)—20 mM phosphate buffer (pH 7), 10:90 *v/v* as the mobile phase, the XBridge C18 column showed a clearly better selectivity for injectable antibiotics such as ceftazidime and ceftriaxone. So, this column was preferred above the Kinetex for further method development.

Some antibiotics, such as ceftazidime, ceftriaxone, and cefepime, are relatively polar compounds that are not very well retained by the C18 column. On the other hand, chloramphenicol, flucloxacillin, and cloxacillin are less polar and showed long retention times. Because of the different characteristics and affinities of the antibiotics for the stationary phase and to limit the total analysis time, gradient elution was applied. Some preliminary gradient elution programs were tested with two different organic modifiers: ACN and MeOH. It was observed that a better separation and more symmetrical peak shapes were obtained using ACN. Moreover, ACN has a lower viscosity, which results in a lower back pressure. Hence, ACN was selected. The chromatographic parameters such as gradient, pH, detection wavelength, flow rate, and column temperature were further optimized in order to develop a screening method for injectable antibiotics with optimal resolution and sharp peaks.

Different proportions of ACN—20 mM phosphate buffer (pH 7.0) were tested. For mobile phase A, ratios of 10:90, 20:80, 6:94, and 5:95 *v*/*v* were tested, whereas for mobile phase B, ratios of 70:30, 60:40, 50:50, 40:60, and 30:70 *v*/*v* were evaluated. It was observed that the best separation of the early eluting peaks (ceftriaxone, ceftazidime, and cefepime) was achieved using 6% ACN in mobile phase A, combined with a gentle gradient with 30% ACN in mobile phase B. The gradient was optimized to obtain baseline separation of the peaks. The gradient started with an isocratic elution of 100% mobile phase A for 2 min, followed by a linear decrease to 60% in 16 min. Then the percentage of mobile phase A further linearly dropped to 0% in 7 min, which was maintained for 10 min. Under these conditions, all of the injectable antibiotics were separated with all resolutions (Rs) ≥ 2. The analysis time was 35 min and so acceptable.

**Figure 2:**
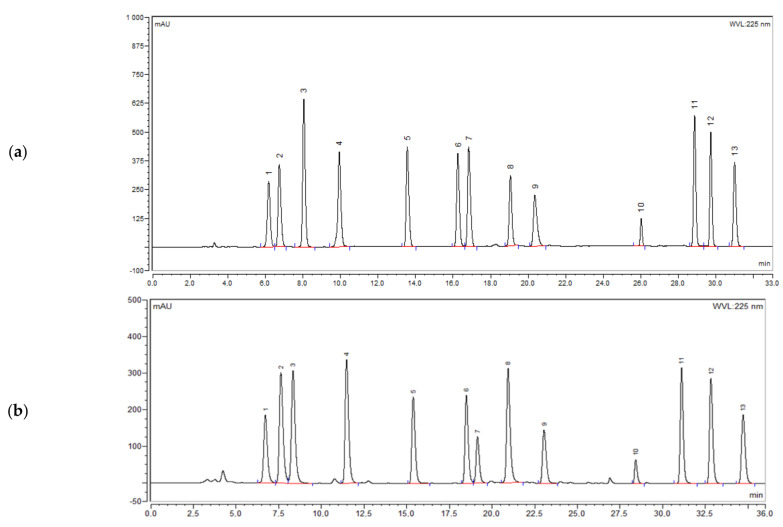
Chromatogram of a mixture of reference standards obtained using (**a**) XBridge C18 column (250 mm × 4.6 mm, 5 µm), injection volume: 20 µL, flow rate: 1 mL/min, and analyte concentrations varying from 0.1 to 0.4 mg/mL; (**b**) XBridge C18 column (250 mm × 3 mm, 5 µm), injection volume: 8 µL, flow rate: 0.4 mL/min, and analyte concentrations varying from 0.04 to 0.16 mg/mL; Peak 1: ceftazidime, 2: ceftriaxone, 3: cefepime, 4: amoxicillin, 5: meropenem, 6: cefotaxime, 7: cefazolin, 8: vancomycin, 9: ampicillin, 10: benzylpenicillin, 11: chloramphenicol, 12: flucloxacillin, and 13: cloxacillin.

Next, the influence of the pH of the buffer solution (3.0, 6.0, 7.0, 7.5, 8.0, 8.5, and 9.0) was evaluated. Increasing somewhat the buffer solution pH improved the selectivity and peak shape. The better separation and peak shape were achieved at pH 8.0. In addition, flow rates of 0.8, 1.0, and 1.2 mL/min and column temperatures of 25, 30, 35, and 40 °C were checked. Optimal separation of the peaks was obtained at a flow rate of 1.0 mL/min and a column temperature of 30 °C. Finally, different detection wavelengths (220, 225, and 230 nm) were examined. Although all antibiotics could be detected at those wavelengths, UV absorbance of benzylpenicillin at 230 nm was relatively weak, and the baseline was most unstable at 220 nm. Hence, a wavelength of 225 nm was chosen, where the signal-to-noise ratio for the benzylpenicillin peak was 1.3 times higher than at 230 nm. Figure 2a shows a chromatogram obtained under the optimized conditions. The quantification method was adopted from the screening method (i.e., the method to screen the identity can also be used for the quantification of injectable antibiotics in formulations).

The method was subsequently evaluated using an XBridge C18 column with a more narrow internal diameter of 3 mm. A chromatogram is depicted in Figure 2b. Most chromatographic conditions were the same, but some adaptations were made, including a flow rate of 0.4 mL/min and an injection volume of 8 µL. The flow rate and the injection volumes were calculated using the equations given in the European Pharmacopoeia [23]. The use of a narrow bore column reduces the mobile phase consumption by 60%. Hence, this method is more economical and ecological. It can be used as well as an alternative for the quality control of injectable antibiotics in developing countries. However, old LC pumps may have problems delivering a stable flow of 0.4 mL/min. Therefore, method validation was performed using the XBridge C18 column with an internal diameter of 4.6 mm.

Compared to the scarce previously published methods for the screening of antibiotics, it is the first time that a method is presented for the 13 compounds studied here. Other papers included less or other antibiotics [24,25] or used more costly equipment [20].

### 2.2. Method Validation

#### 2.2.1. Specificity

The specificity of the method was evaluated by comparing the chromatograms of the standards with those of commonly used excipients for injectable antibiotics. There was no interference from excipients at the retention times of the analytes. Moreover, all peaks corresponding to the antibiotics were baseline separated from one another (Rs ≥ 2) (Figure 2a). Therefore, the method is specific and selective for the antibiotics.

#### 2.2.2. Robustness

**Figure 3:**
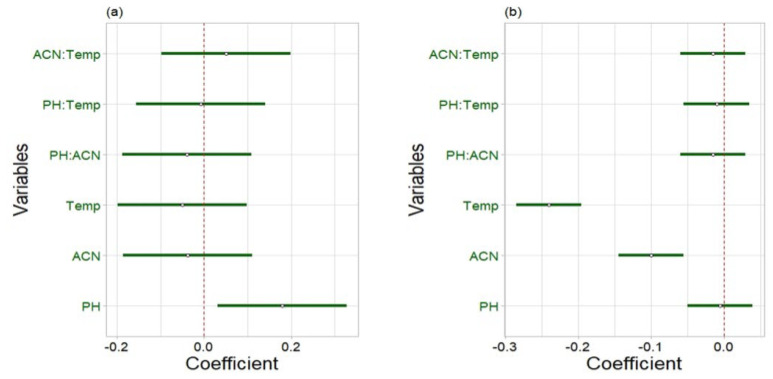
Regression coefficient plots for resolution between (**a**) ceftazidime–ceftriaxone and (**b**) cefotaxime–cefazolin.

Three factors were evaluated: column temperature (Temp), mobile phase pH, and the percentage of acetonitrile (% ACN) in mobile phase B. In the regression coefficient plots (Figure 3), variables were considered not significant if zero (indicated by the red dashed line) was included in their 95% confidence interval (CI) (indicated by the green lines). This statistical analysis revealed that, for the ceftazidime–ceftriaxone peak pair, only the buffer pH significantly affected the resolution, showing a positive effect. This means that with higher pH values, the resolution enhances (and vice versa). On the other hand, for the cefotaxime–cefazolin peak pair, lower temperature and lower percentage of ACN improved the resolution, as indicated by the significant negative effects. Other parameters or interactions were found to be not significant.

**Figure 4:**
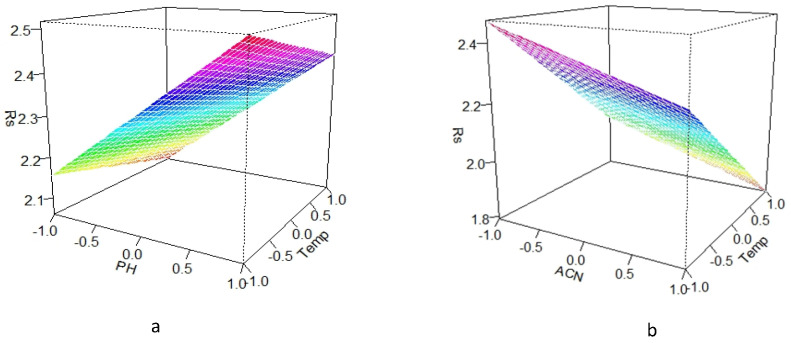
Response surface plots indicating the effect of (**a**) buffer pH and column temperature on the resolution between the ceftazidime–ceftriaxone peaks and (**b**) column temperature and % ACN in mobile phase B on the resolution between the cefotaxime–cefazolin peaks.

The effect of parameters with a significant influence on the resolution of the ceftazidime–ceftriaxone and cefotaxime–cefazolin peaks was further explored by the response surface plots. These revealed that the resolution between the ceftazidime–ceftriaxone peaks within the experimental domain was at least 2.0, whereas the resolution between cefotaxime and cefazolin was always above 1.8 (Figure 4). So, even at the worst conditions tested, both pairs remained almost baseline separated. This indicates that the method is robust. Hence, no problems are expected when the parameter conditions vary somewhat.

#### 2.2.3. Linearity

Calibration curves were constructed by plotting the peak areas *versus* the respective standard concentrations. After fitting the equation *y* = m*x* + b (with *y*: peak area, *x*: concentration in mg/mL, m: slope, and b: intercept), the determination coefficients (R^2^) for all injectable antibiotics were above 0.999, indicating good correlation. The residual plots were also randomly scattered around the horizontal zero axis. For all equations, zero was included in the 95% CI of the intercept.

Depending on the label claim of commercial samples, solutions should be prepared with expected concentrations within the validated range.

#### 2.2.4. Accuracy

The accuracy of the method was expressed as percent recovery. The percentage recoveries are shown in Table 1. The results were all in the range of 98.2–101.6% and so within the acceptance limits of 98.0–102.0%. Thus, the developed method was found to be accurate.

#### 2.2.5. Precision

Precision was evaluated based on repeatability (intraday precision) and interday precision. It was expressed based on the relative standard deviation (RSD) of the peak areas. As shown in Table 1, the RSD values were in the range of 0.1–0.5% and 0.1–0.9% for intraday and interday precision, respectively.

#### 2.2.6. Sensitivity

The results for the limit of detection (LOD) and limit of quantification (LOQ) are presented in Table 2. Although those values are maybe less important regarding the aim of this study, it is good to have an indication of the sensitivity of the method.

### 2.3. Analysis of Commercial Samples

The optimized method was successfully applied to analyze injectable antibiotics in commercial samples from Ethiopia. The United States Pharmacopeia (USP) [26] specifies that the percentage content of API in the injection formulations of ceftazidime, meropenem, and cloxacillin should fall within the range of 90.0–120.0%, while for vancomycin, ceftriaxone, ampicillin, cefotaxime, and cefepime, the range is 90.0–115.0%. The identification test results confirmed the presence of the APIs in all injections. Furthermore, the assay results for the tested samples were between 90.4 and 106.9% (Table 3). Hence, all samples complied with the USP requirements for assay. Although nearly half of the samples showed an active content between 90 and 95% of the labeled amount, they still passed the USP assay criteria and so comply with the specifications. Chromatograms of commercial samples are shown in the Supplementary Material (Figure S1).

## 3. Materials and Methods

### 3.1. Reagents and Materials

The reagents used in this study were HPLC-grade ACN (Acros Organics, Geel, Belgium), monobasic potassium phosphate (99.5%, VWR, Leuven, Belgium), L (+)-arginine (98%, AppliChem, Darmstadt, Germany), sodium carbonate (99.8%, Sigma Aldrich, Steinheim, Germany), and sodium hydroxide (VWR, Leuven, Belgium). A Milli-Q water purification system (Millipore, Bedford, MA, USA) was used to prepare ultrapure water. Chromafil syringe filters (0.45 µm) were from Carl Roth (Karlsruhe, Germany). Reference standards for cefazolin, ampicillin (sodium), cefotaxime (sodium), cefepime (dihydrochloride monohydrate), amoxicillin (trihydrate), chloramphenicol, ceftazidime, ceftriaxone (sodium), vancomycin (hydrochloride), flucloxacillin (sodium), cloxacillin (sodium), benzylpenicillin (sodium), and meropenem (trihydrate) were from the European Directorate for the Quality of Medicines and HealthCare (Strasbourg, France). Seventeen injectable samples, involving eight different antibiotics, were randomly collected from community pharmacies in Ethiopia (Table 4). As mentioned in the respective leaflets, the cefepime samples also contained arginine, and the ceftazidime samples also contained sodium carbonate. All samples were analyzed before their expiry date.

### 3.2. Chromatographic Conditions

An Ultimate 3000 HPLC system from Dionex (Sunnyvale, CA, USA) with a quaternary pump, autosampler, and UV–visible detector was used. The equipment and output were monitored by Chromeleon software 6.80 (Dionex). Initially, an XBridge C18 column (250 mm × 4.6 mm, 5 µm) (Waters, Wexford, Ireland) and a Kinetex C18 column (150 mm × 4.6 mm, 2.6 µm) (Phenomenex, Torrance, CA, USA) were evaluated. The optimum separation of the injectable antibiotics was obtained in gradient mode, using the XBridge C18 column (250 mm × 4.6 mm, 5 µm). An XBridge C18 column (250 mm × 3 mm, 5 µm) with a more narrow internal diameter was also evaluated as an alternative. For both columns, the same mobile phase composition, gradient program, detection wavelength, and column temperature were used. Mobile phases A and B consisted of ACN—20 mM phosphate buffer (pH 8.0, adjusted with 2 M sodium hydroxide) in ratios of 6:94 (*v*/*v*) and 30:70 (*v*/*v*), respectively. The gradient program was: 0–2 min, 0% B; 2–18 min, 0–40% B; 18–25 min, 100% B; 25–35 min, 100% B, followed by approximately 3 min for system re-equilibration. A detection wavelength of 225 nm was used, and the column temperature was maintained at 30 °C. For the XBridge C18 column with a diameter of 4.6 mm, a flow rate of 1 mL/min and an injection volume of 20 μL were used, while for this with a diameter of 3 mm, an injection volume of 8 μL and a flow rate of 0.4 mL/min were used for the separation of the injectable antibiotics.

### 3.3. Screening Method

#### 3.3.1. Preparation of Standard Solutions

Reference standards of ceftazidime, ceftriaxone, cefotaxime, vancomycin, flucloxacillin, cloxacillin (10 mg each), cefepime, amoxicillin, cefazolin, ampicillin, benzylpenicillin, chloramphenicol (20 mg each), and meropenem (40 mg) were dissolved in 100 mL of mobile phase A. This mixture was found to be stable (all compounds > 95%) for one month when stored at −20 °C.

#### 3.3.2. Preparation of Sample Solutions

The samples were obtained as powder filled in vials. Before the powder was removed, the complete vial was weighed. After washing and drying, the empty vial was weighed. The difference in weight was used to calculate the total weight of the powder in the vial. Stock solutions were prepared by dissolving an equivalent of 20 mg of cefepime, ampicillin, ceftazidime, ceftriaxone, cefotaxime, vancomycin, or cloxacillin, or 40 mg of meropenem in 20 mL of mobile phase A. Two milliliters of the individual stock solutions of ceftazidime, ceftriaxone, cefotaxime, vancomycin, or cloxacillin and 4 mL of the individual stock solutions of cefepime, ampicillin, and meropenem were further diluted to 20 mL with mobile phase A. These solutions were filtered through a 0.45 µm syringe filter (Chromafil, Carl Roth, Karlsruhe, Germany).

### 3.4. Quantification Method

#### 3.4.1. Preparation of Standards

Twenty milligrams of references of cefepime, ampicillin, ceftazidime, ceftriaxone, cefotaxime, vancomycin, cloxacillin, and 40 mg of meropenem were each dissolved in 20 mL of mobile phase A. Two milliliters of the standard solutions of ceftazidime, ceftriaxone, cefotaxime, vancomycin, or cloxacillin and 4 mL of the standard solutions of cefepime, ampicillin, and meropenem were further diluted with 20 mL of mobile phase A. The diluted solution of each antibiotic was separately injected into the LC system. The solutions were found to be stable (>99%) for at least one week when kept at −20 °C.

#### 3.4.2. Preparation of Sample Solutions

The same procedures were followed as outlined in Section 3.3.2.

### 3.5. Validation of the Screening and Quantification Method

The method was validated per the International Council for Harmonization (ICH) guidelines [27], evaluating specificity and robustness for the identity screening and linearity, precision, accuracy, and sensitivity for the quantification method.

Specificity was evaluated by injecting common excipients found in injectable antibiotics, such as sodium carbonate and arginine, into the LC. These excipients were selected based on the manufacturer’s information of the commercial injectable products collected from Ethiopia. In case products should be analyzed that contain excipients that were not included in this study, it is advisable to check for possible interference. Moreover, the method’s specificity was further evaluated by verifying that all antibiotics were separated from one another.

For the robustness study, an experimental design using R software version 4.1.3 (R Studio, Boston, MA, USA) was used. The influence of three chromatographic factors on the resolution of the most critical peak pairs (ceftazidime–ceftriaxone and cefotaxime–cefazolin) was evaluated using a two-level full factorial design. The investigated parameters with their lower and upper levels can be found in Table 5. A total of 2*^k^* + *n* experiments (i.e., 11 experiments including the central points) were performed, where *k* is the number of factors and *n* is the number of times that the center point was repeated (*n* = 3).

The following equation was used to mathematically describe the relation between the response (y) and the experimental variables (*x_i_*, *x_j_*, ...): *y = b*_0_ *+ b_i_x_i_ + b_j_x_j_ + b_ij_x_i_ x_j_ + ··· + ε*, where the experimental error is represented by *ε* and the regression coefficients are represented by the *b*-values with *b*_0_ the intercept, *b_i_* and *b_j_* describe the quantitative effect of the variables *x_i_* and *x_j_*, respectively, and *b_ij_* represents the interaction effect between both variables.

Linearity of the method was tested in the concentration ranges of 0.06–0.14 mg/mL for ceftazidime, ceftriaxone, cefotaxime, vancomycin, flucloxacillin, and cloxacillin; 0.12–0.28 mg/mL for cefepime, amoxicillin, cefazolin, ampicillin, benzylpenicillin, and chloramphenicol; and 0.24–0.56 mg/mL for meropenem. Calibration curves were established using five concentration levels: 60, 80, 100, 120, and 140% of the standard concentrations. These solutions were analyzed in triplicate. The intercept, residuals, and R^2^ values of the calibration curves were assessed.

The intraday precision was evaluated by six injections of 100% of the standard concentrations on the same day. The interday precision was determined from 18 injections of the same concentration over three consecutive days.

The accuracy of the method was determined by spiking reference standards of each antibiotic in a blank excipient mixture containing arginine and sodium carbonate. These excipients were present in cefepime and ceftazidime injection samples, respectively. Triplicate determinations were performed at three concentration levels (i.e., 80%, 100%, and 120%).

For sensitivity, LOD and LOQ values were determined by serial dilution of the standard solution of each antibiotic to obtain signal-to-noise (S/N) ratios of 3 and 10, respectively.

### 3.6. Application of the Method

The method was applied to analyze 17 injectable antibiotic samples from Ethiopia. The identity was checked, and an assay was performed in triplicate, and the percentage content of the respective drug in the samples was calculated.

## 4. Conclusions

A novel LC-UV method was developed for both screening and quantification of 13 injectable antibiotics. The analysis time was only 35 min, and peaks could be identified based on their retention times. No interference from known excipients was observed. The developed method demonstrated good specificity, sensitivity, linearity, accuracy, precision, and robustness. The method uses conventional LC-UV equipment, so it is affordable for routine quality control in low-income countries. The availability of a single method for multiple antibiotics is clearly more cost-effective and practical compared to individual methods for each antibiotic. The proposed method was successfully applied to analyze 17 injectable antibiotics collected from the Ethiopian market. All samples complied for identification and met the USP requirements for assay, although several samples were close to the lower limit of 90%.

## Figures and Tables

**Table 1:** Accuracy and precision results.

Analytes	Intraday Precision (% RSD), *n* = 6	Interday Precision (% RSD), *n* = 18	% Recovery (% RSD), *n* = 3		
			80%	100%	120%
Ceftazidime	0.3	0.3	100.3 (0.2)	99.4 (0.1)	98.9 (0.2)
Ceftriaxone	0.1	0.1	101.6 (0.4)	99.9 (0.1)	100.8 (0.3)
Cefepime	0.5	0.5	99.4 (0.4)	98.2 (0.2)	98.3 (0.3)
Amoxicillin	0.1	0.3	101.3 (0.1)	100.8 (0.1)	100.0 (0.1)
Meropenem	0.4	0.9	101.5 (0.3)	98.8 (0.2)	99.6 (0.2)
Cefotaxime	0.4	0.7	101.5 (0.2)	99.0 (0.2)	99.9 (0.2)
Cefazolin	0.1	0.2	99.9 (0.4)	99.6 (0.5)	99.6 (0.1)
Vancomycin	0.2	0.5	99.8 (0.3)	99.1 (0.1)	100.2 (0.1)
Ampicillin	0.3	0.3	101.2 (0.4)	99.4 (0.5)	99.9 (0.1)
Benzylpenicillin	0.2	0.6	100.0 (0.4)	100.2 (0.1)	99.8 (0.1)
Chloramphenicol	0.1	0.8	100.1 (0.2)	100.1 (0.1)	100.5 (0.1)
Flucloxacillin	0.2	0.7	99.5 (0.3)	99.7 (0.1)	99.5 (0.1)
Cloxacillin	0.4	0.8	99.4 (0.3)	99.5 (0.1)	99.7 (0.2)

**Table 2:** LOD and LOQ of injectable antibiotics.

Analytes	LOQ (µg/mL)	LOD (µg/mL)
Ceftazidime	0.018	0.005
Ceftriaxone	0.018	0.005
Cefepime	0.039	0.012
Amoxicillin	0.053	0.016
Meropenem	0.174	0.052
Cefotaxime	0.059	0.018
Cefazolin	0.242	0.072
Vancomycin	0.216	0.065
Ampicillin	0.592	0.178
Benzylpenicillin	0.735	0.221
Chloramphenicol	0.391	0.117
Flucloxacillin	0.068	0.020
Cloxacillin	0.144	0.043

**Table 3:** Assay results of injectable antibiotics.

Analytes	Sample Code	% Content (% RSD)
Vancomycin 1 g	Sample 1	106.9 (0.9)
	Sample 2	93.7 (0.9)
	Sample 3	104.1 (0.8)
Ceftazidime 1 g	Sample 4	99.2 (0.9)
	Sample 5	100.7 (0.7)
Ceftriaxone 1 g	Sample 6	98.5 (0.1)
	Sample 7	99.1 (0.2)
	Sample 8	97.9 (0.7)
	Sample 9	95.9 (0.8)
Cefepime 1 g	Sample 10	90.7 (0.3)
	Sample 11	92.5 (0.7)
	Sample 12	91.5 (0.8)
	Sample 13	90.4 (0.4)
Meropenem 1 g	Sample 14	91.8 (0.2)
Cloxacillin 500 mg	Sample 15	101.3 (0.3)
Ampicillin 1 g	Sample 16	94.6 (0.4)
Cefotaxime 1 g	Sample 17	92.8 (0.8)

**Table 4:** Injectable antibiotic samples included in this study.

Analytes	Sample Code	Country of Origin
Vancomycin 1 g	Sample 1	India
	Sample 2	India
	Sample 3	India
Ceftazidime 1 g	Sample 4	India
	Sample 5	India
Ceftriaxone 1 g	Sample 6	India
	Sample 7	India
	Sample 8	India
	Sample 9	China
Cefepime 1 g	Sample 10	India
	Sample 11	India
	Sample 12	India
	Sample 13	Ethiopia
Meropenem 1 g	Sample 14	China
Cloxacillin 500 mg	Sample 15	China
Ampicillin 1 g	Sample 16	China
Cefotaxime 1 g	Sample 17	India

**Table 5:** Chromatographic parameters and their levels used in experimental design.

Parameter	Lower Level (−)	Central Level (0)	Higher Level (+)
Buffer pH	7.8	8	8.2
% ACN in mobile phase B	28	30	32
Column temperature (°C)	28	30	32

## Data Availability

Data are contained within this article.

## Supplementary Materials

The following supporting information can be downloaded at: https://www.mdpi.com/article/10.3390/molecules30102151/s1, Figure S1: Chromatograms of commercial samples of injectable antibiotics collected from the Ethiopian mar-25 ket.
