# Supplementary material for: A Cost-Effective Liquid Chromatography Method with Ultraviolet Detection for Identity Screening and Assay of Injectable Antibiotics
Source: Molecules. 2025 May 13;30(10):2151. doi: 10.3390/molecules30102151 (PMC12114479; doi:10.3390/molecules30102151)
Supplement: Supplementary file 1 [file molecules-30-02151-s001.zip › molecules-3578085-supplementary.pdf]

molecules

MDPI

*Supplementary material*

# A cost-effective LC-UV method for identity screening and assay of injectable antibiotics

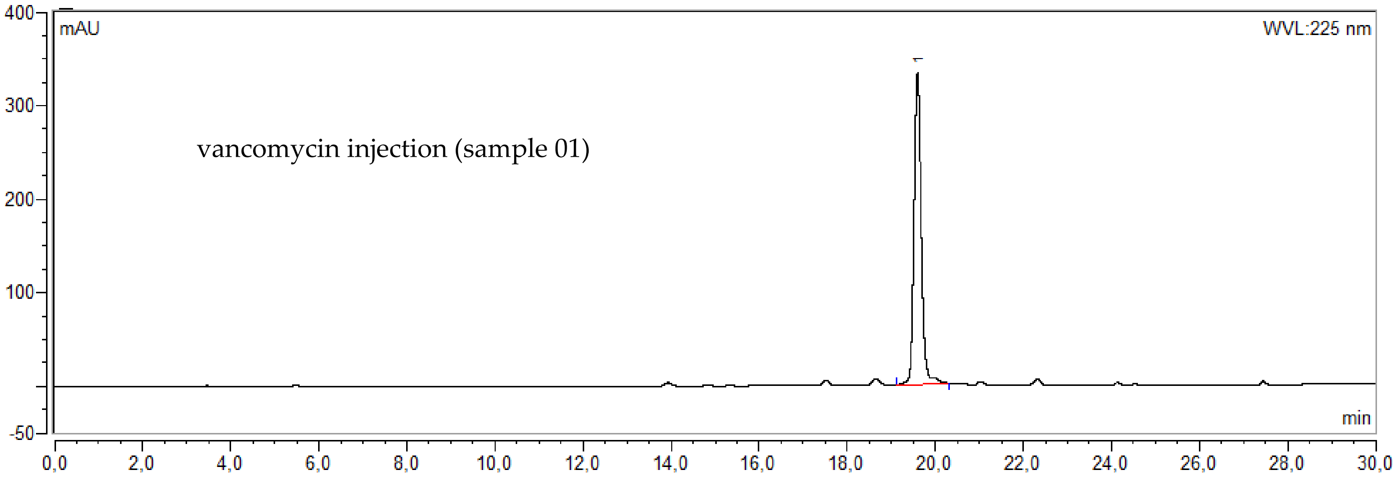


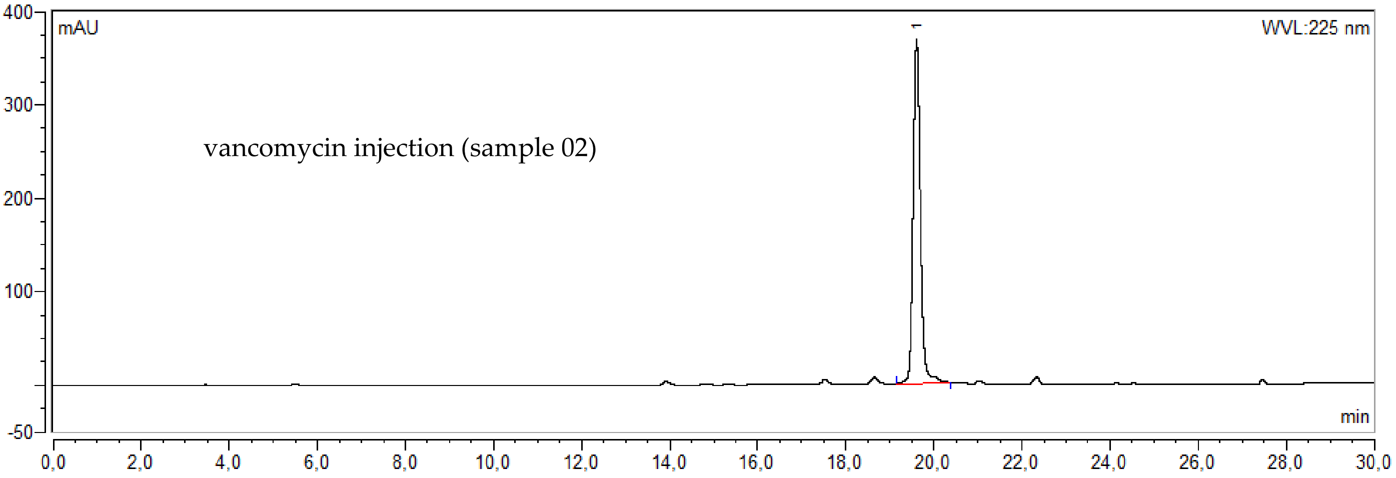


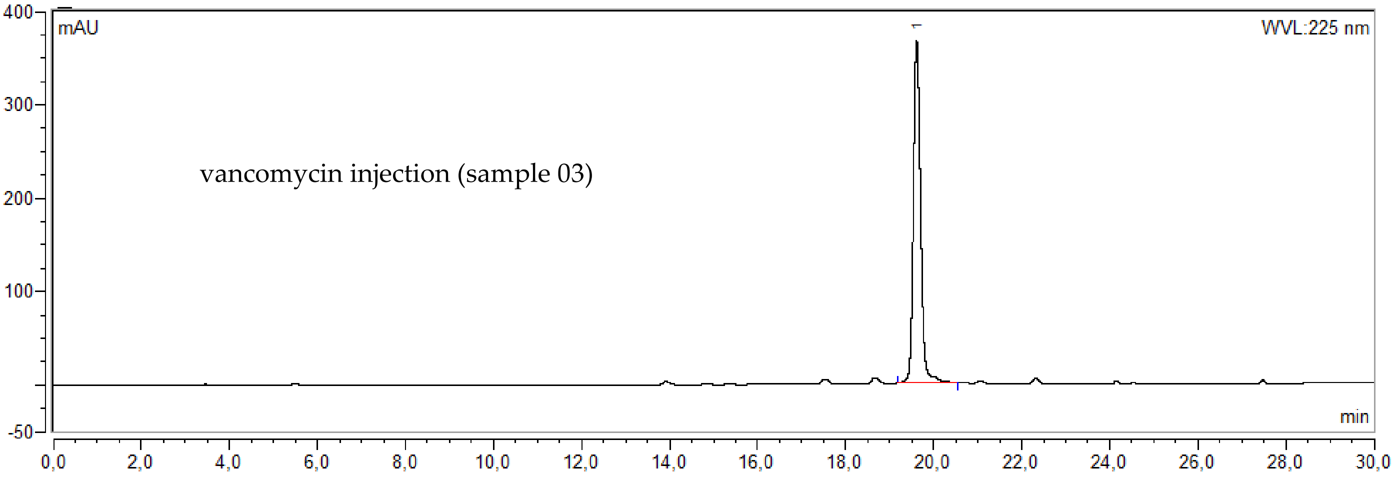


 https://doi.org/10.3390/xxxxx

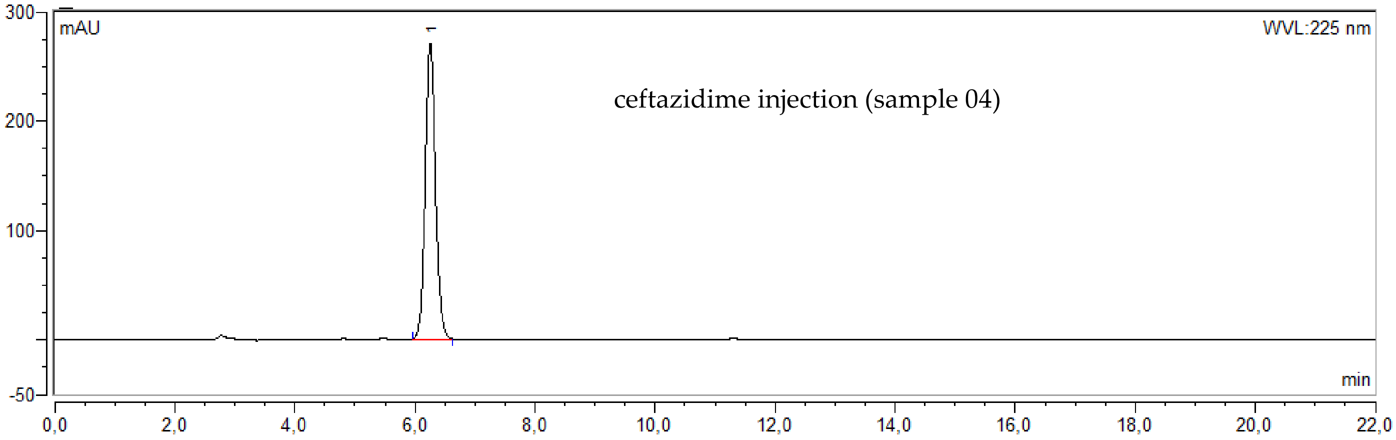
mAU
WVL:225 nm
ceftazidime injection (sample 04)
300
200
100
-50
min
0,0
2,0
4,0
6,0
8,0
10,0
12,0
14,0
16,0
18,0
20,0
22,0

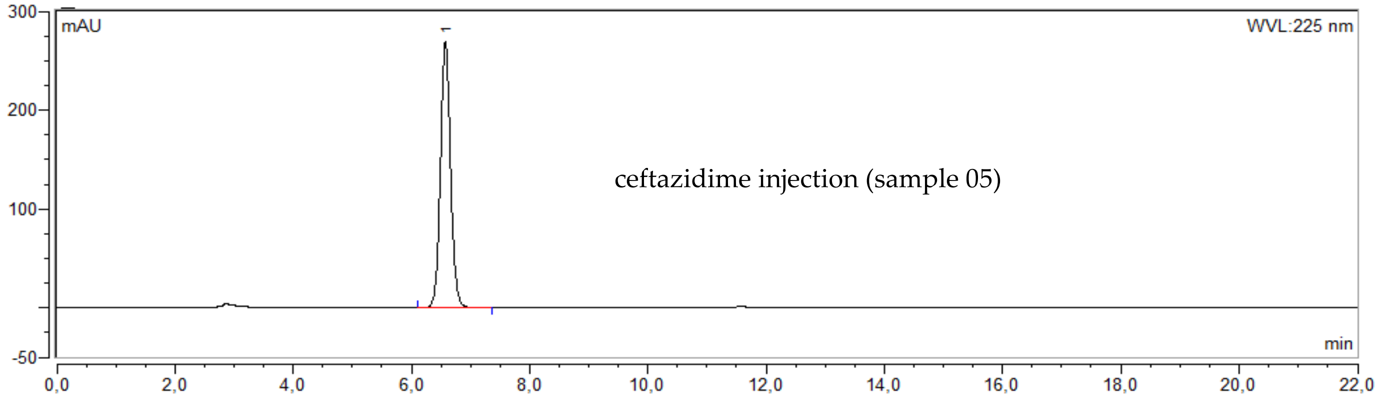
mAU
WVL:225 nm
ceftazidime injection (sample 05)
300
200
100
-50
min
0,0
2,0
4,0
6,0
8,0
10,0
12,0
14,0
16,0
18,0
20,0
22,0

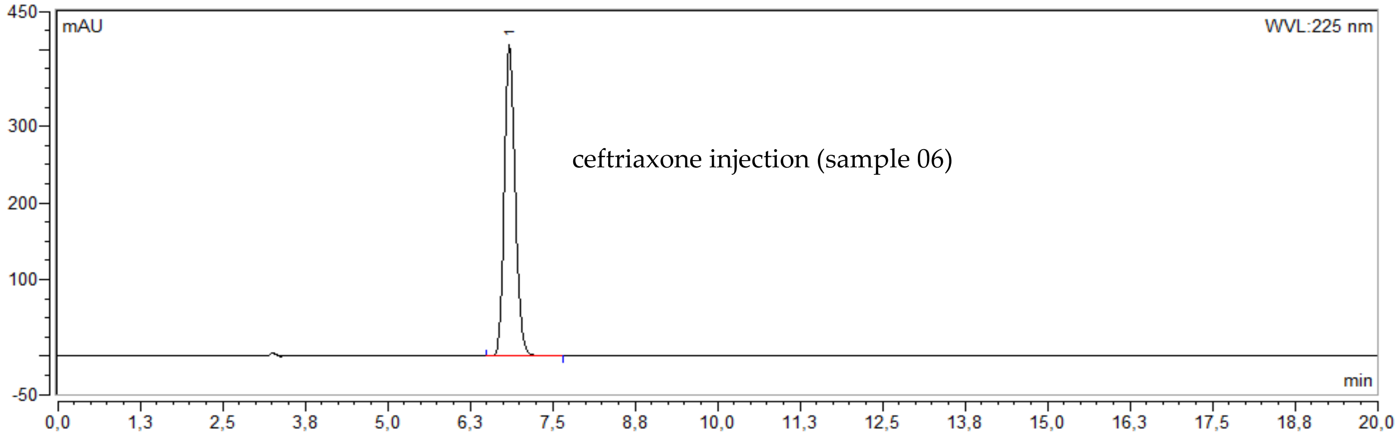
mAU
WVL:225 nm
ceftriaxone injection (sample 06)
min
450
300
200
100
-50
0,0
1,3
2,5
3,8
5,0
6,3
7,5
8,8
10,0
11,3
12,5
13,8
15,0
16,3
17,5
18,8
20,0

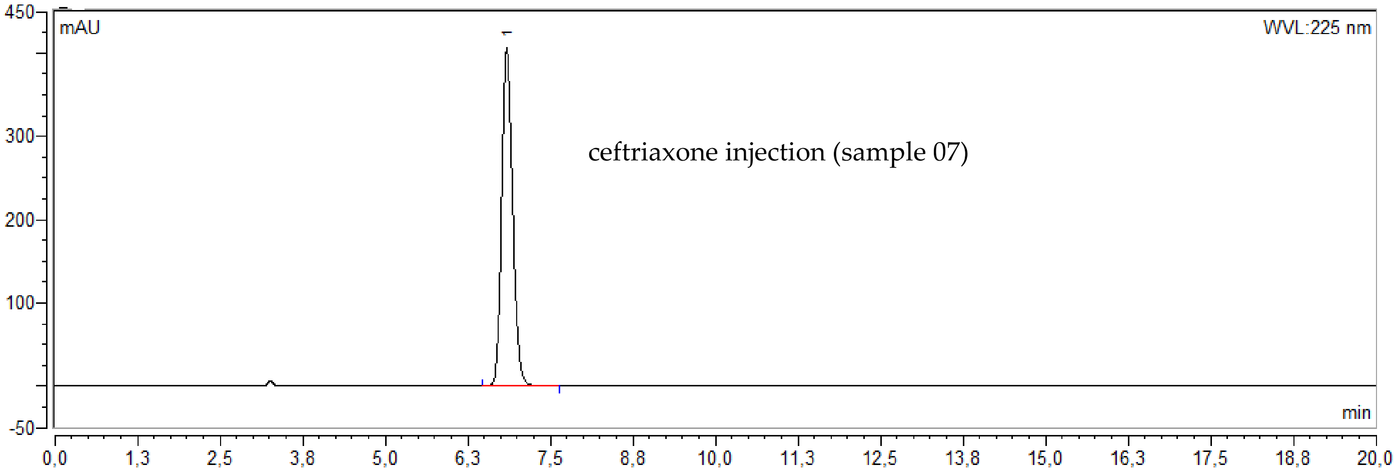
mAU
WVL:225 nm
ceftriaxone injection (sample 07)
min
450
300
200
100
-50
0,0
1,3
2,5
3,8
5,0
6,3
7,5
8,8
10,0
11,3
12,5
13,8
15,0
16,3
17,5
18,8
20,0

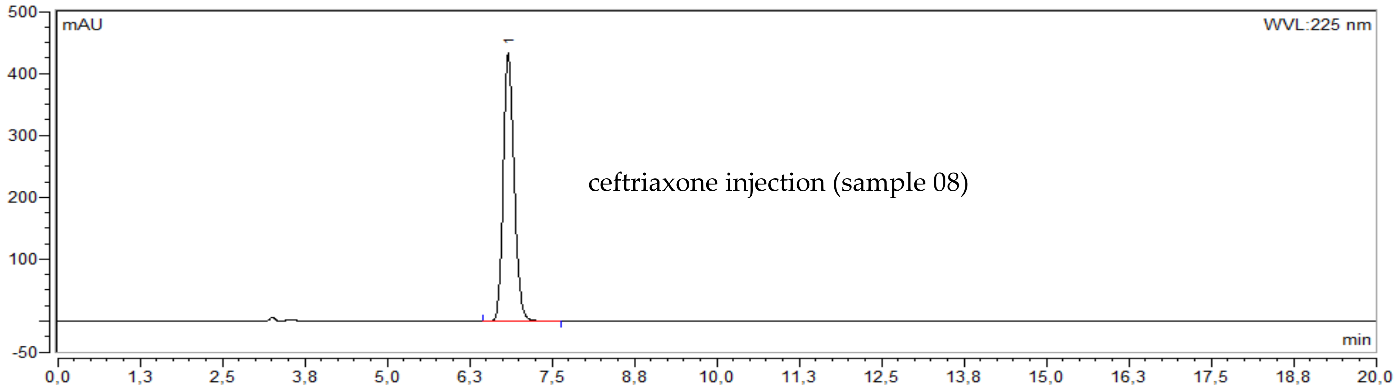
mAU
WVL:225 nm
ceftriaxone injection (sample 08)
min
500
400
300
200
100
-50
0,0
1,3
2,5
3,8
5,0
6,3
7,5
8,8
10,0
11,3
12,5
13,8
15,0
16,3
17,5
18,8
20,0

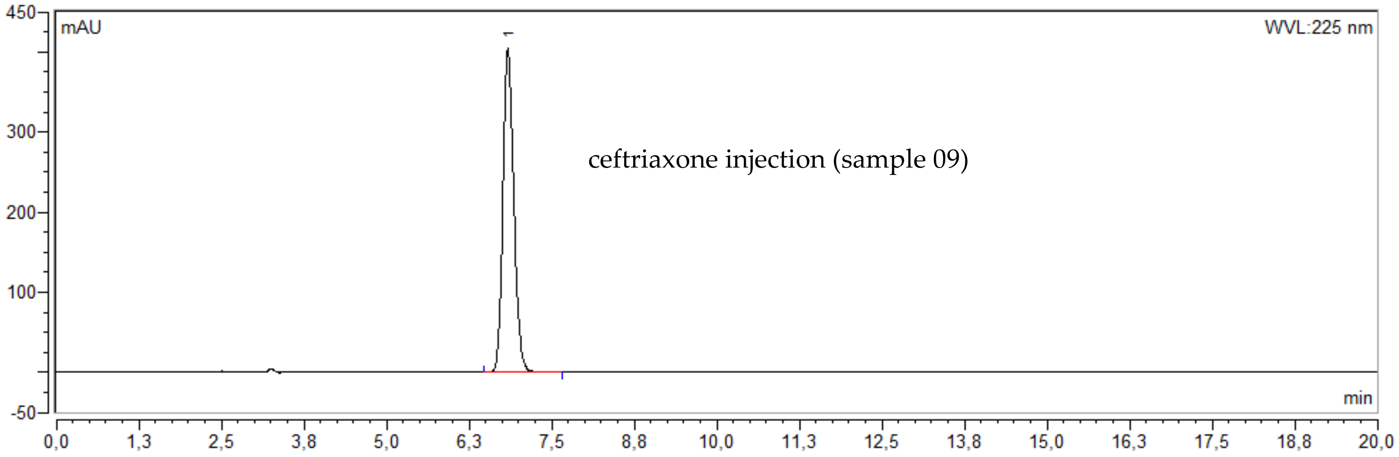
mAU
WVL:225 nm
ceftriaxone injection (sample 09)
min
450
300
200
100
-50
0,0
1,3
2,5
3,8
5,0
6,3
7,5
8,8
10,0
11,3
12,5
13,8
15,0
16,3
17,5
18,8
20,0

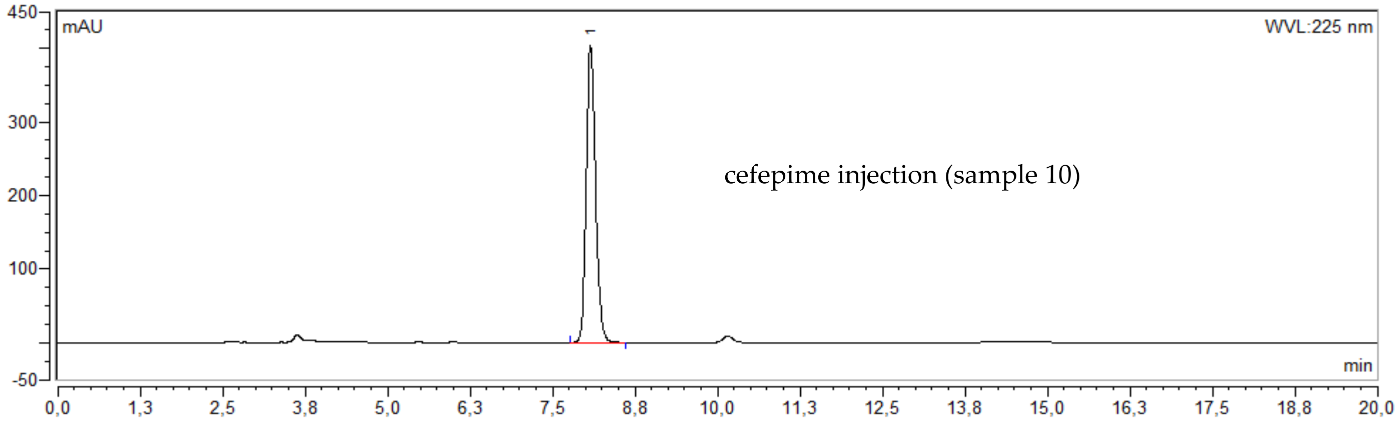
mAU
WVL:225 nm
cefepime injection (sample 10)
450
300
200
100
-50
min
0,0 1,3 2,5 3,8 5,0 6,3 7,5 8,8 10,0 11,3 12,5 13,8 15,0 16,3 17,5 18,8 20,0

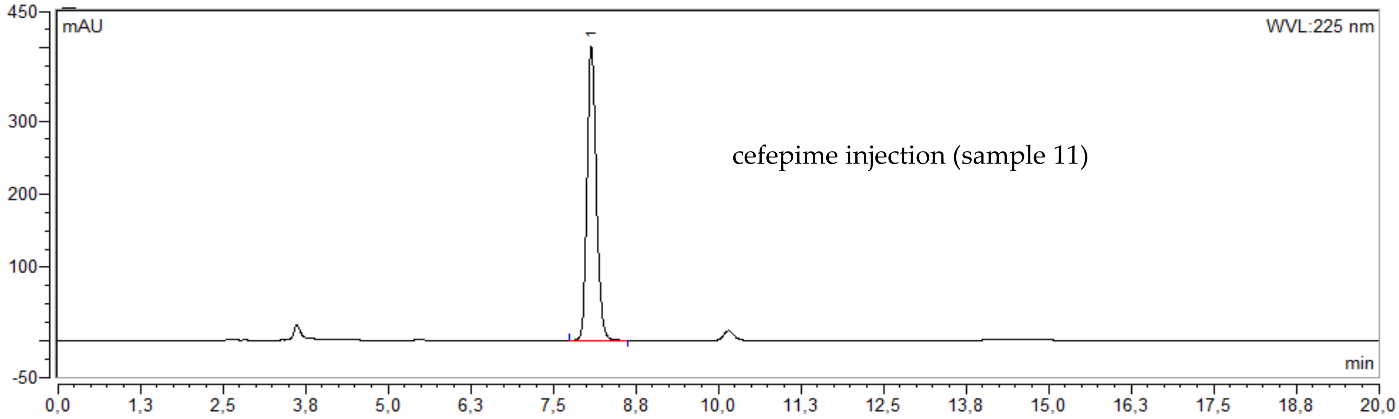
mAU
WVL:225 nm
cefepime injection (sample 11)
450
300
200
100
-50
min
0,0 1,3 2,5 3,8 5,0 6,3 7,5 8,8 10,0 11,3 12,5 13,8 15,0 16,3 17,5 18,8 20,0

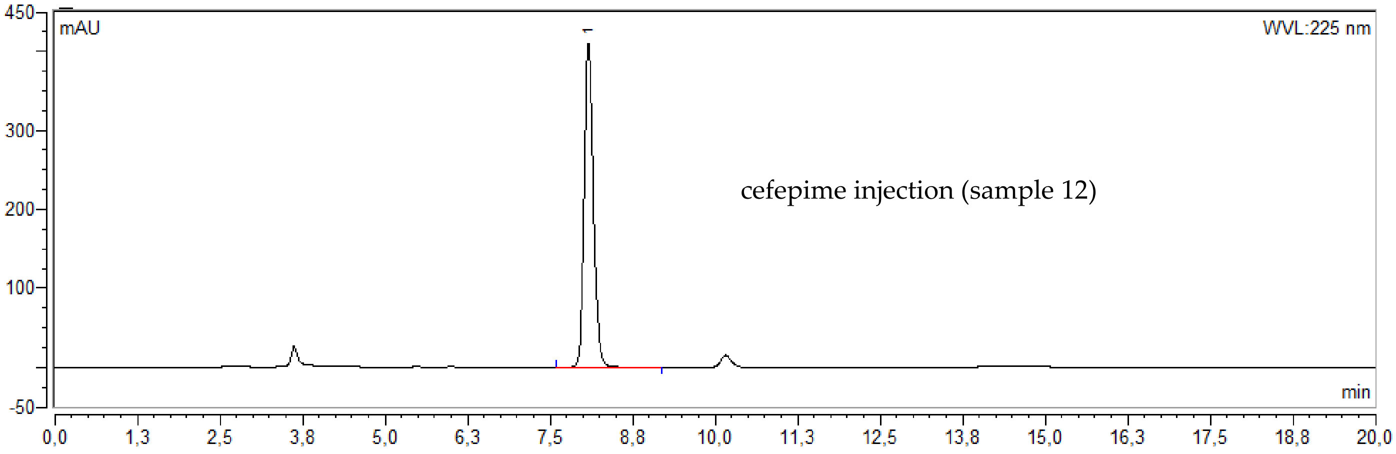
mAU
WVL:225 nm
cefepime injection (sample 12)
450
300
200
100
-50
min
0,0 1,3 2,5 3,8 5,0 6,3 7,5 8,8 10,0 11,3 12,5 13,8 15,0 16,3 17,5 18,8 20,0

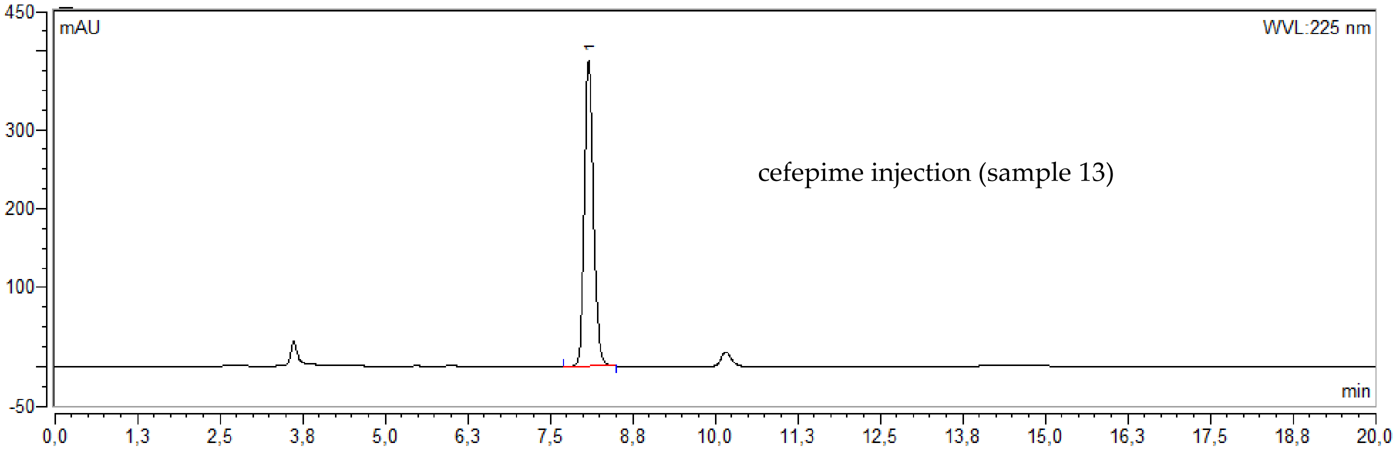
mAU
WVL:225 nm
cefepime injection (sample 13)
450
300
200
100
-50
min
0,0 1,3 2,5 3,8 5,0 6,3 7,5 8,8 10,0 11,3 12,5 13,8 15,0 16,3 17,5 18,8 20,0

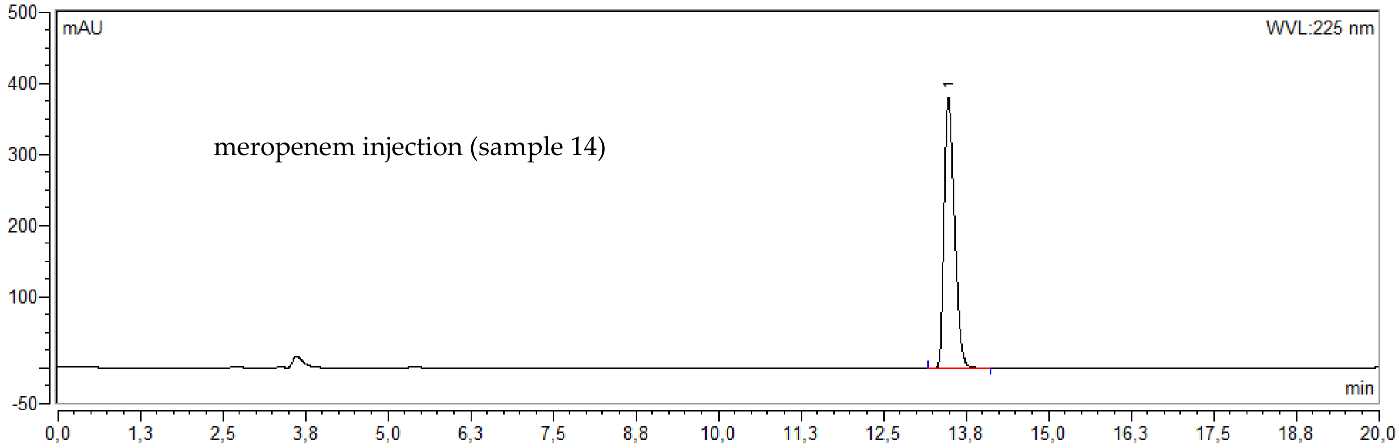
mAU
WVL:225 nm
meropenem injection (sample 14)
500
400
300
200
100
-50
min
0,0
1,3
2,5
3,8
5,0
6,3
7,5
8,8
10,0
11,3
12,5
13,8
15,0
16,3
17,5
18,8
20,0

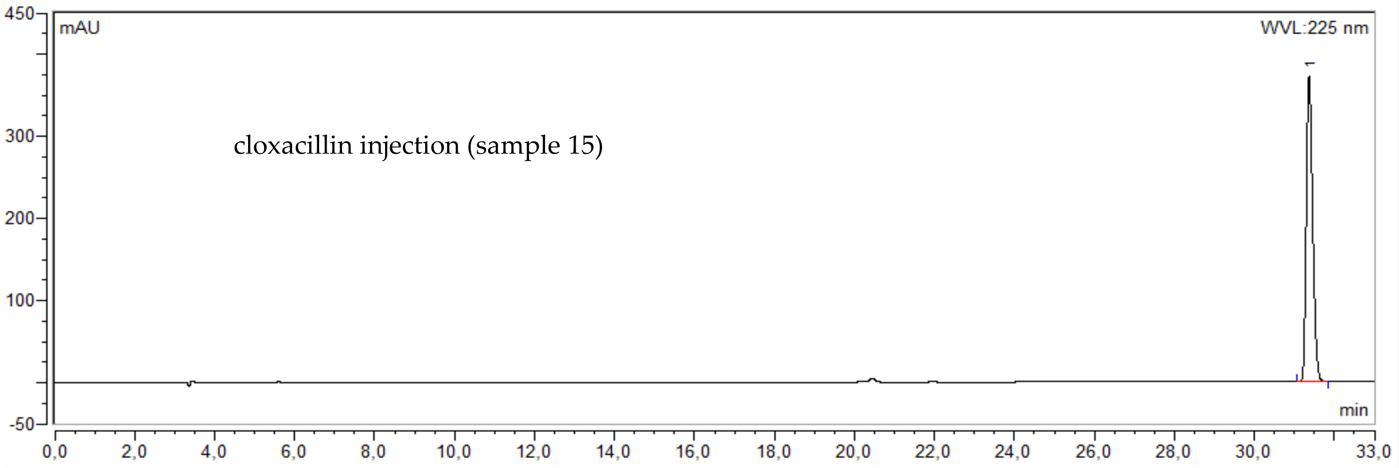
mAU
WVL:225 nm
cloxacillin injection (sample 15)
450
300
200
100
-50
min
0,0
2,0
4,0
6,0
8,0
10,0
12,0
14,0
16,0
18,0
20,0
22,0
24,0
26,0
28,0
30,0
33,0

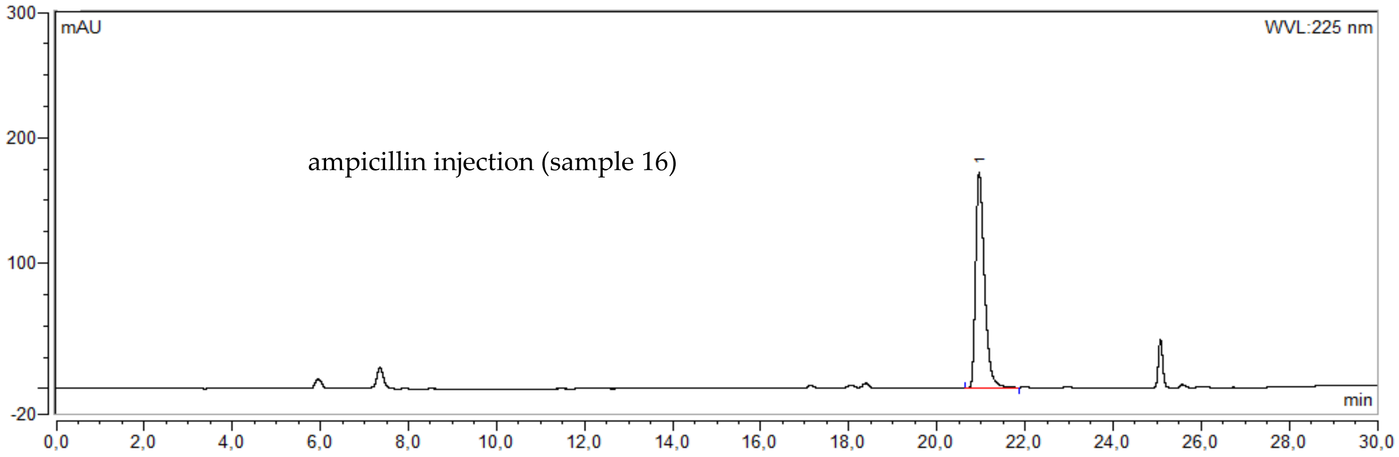
mAU
WVL:225 nm
ampicillin injection (sample 16)
300
200
100
-20
min
0,0
2,0
4,0
6,0
8,0
10,0
12,0
14,0
16,0
18,0
20,0
22,0
24,0
26,0
28,0
30,0

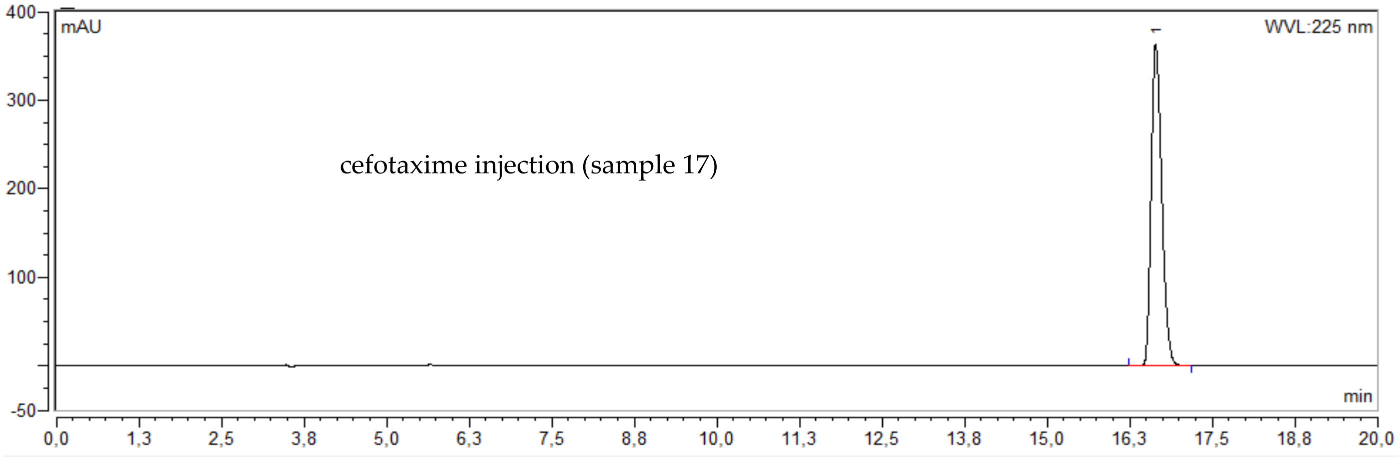


**Figure S1.** Chromatograms of commercial samples of injectable antibiotics collected from the Ethiopian market
